# Adherence to Hypoglycemic Agents in Type 2 Diabetes Mellitus: A Cross-Sectional Study

**DOI:** 10.7759/cureus.22626

**Published:** 2022-02-26

**Authors:** Yara A Khayyat, Reem M Alshamrani, Doha M Bintalib, Najwa A Alzahrani, Sulafa Alqutub

**Affiliations:** 1 Family Medicine, Ministry of Health, Jeddah, SAU; 2 Family and Community Medicine, University of Jedda, Jeddah, SAU

**Keywords:** medication, determinants, adherence, oral hypoglycemic agent, type 2 diabetes

## Abstract

Aims

This study aimed to elucidate the level and determinants of adherence to oral hypoglycemic agents (OHAs) among type 2 diabetes mellitus patients and to employ patient interview as a prediction tool for suboptimal adherence, for preventing and reducing complications.

Methods

In this analytical, cross-sectional study, 383 patients with type 2 diabetes mellitus were interviewed using an electronic, self-constructed, validated questionnaire. Patients were recruited from all Ministry of Health centers across Jeddah, through stratified random sampling. Univariate and multivariate logistic regression analyses were used to evaluate the significance of the results.

Results

Suboptimal levels of adherence were reported by 74.9% of the participants. Predictors of suboptimal adherence are as follows: younger age (P = 0.003), employment [odd ratio (OR), 1.7; 95% confidence interval (CI), 1.1-3.0], unavailability of reminder (OR, 1.9; 95% CI, 1.1-3.1), and non-commitment to appointments (OR, 6.1; 95% CI, 1.1-3.1).

Conclusion

The level of adherence to OHAs was found to be suboptimal. Encountering any of the predictors of suboptimal adherence while interviewing the patient should prompt extra vigilance in the approach. Furthermore, utilizing methods to augment adherence might be prudent.

## Introduction

Diabetes mellitus (DM) is recognized as a genuine threat to public health [1]. By definition, diabetes is “a chronic, metabolic disease characterized by elevated level of blood glucose (or blood sugar), which leads over time to serious damage to heart, blood vessels, eyes, kidneys and nerves” [2]. This damage is often represented by complications, such as coronary artery disease, peripheral artery disease, stroke, blindness, renal impairment, and nerve damage [3]. The prevalence of diabetes among adults in Saudi Arabia is experiencing a rapid rise, it is expected that the total number of cases will reach around 7.5 million by 2035 [4]. Worldwide, diabetes mortality reached five million deaths, which outweighed the combined mortality of HIV/AIDS, tuberculosis, and malaria [5]. In fact, DM has been determined as the sixth leading cause of death in Saudi Arabia [6].

Adherence is indispensable for the successful management of DM [7]. If a patient does not implement the agreed-upon therapeutic plan, the treatment is rendered obsolete. Securing adherence is equivalent to securing the first step on a path leading away from complications [8].

According to the World Health Organization (WHO), the average rate of medication adherence is only 50% in developed countries. As for developing countries, it is estimated to be even lower [9].

In Saudi Arabia, the levels of adherence to oral hypoglycemic agents (OHA) differ from one region to another. Nevertheless, a suboptimal level of adherence prevails [10-13].

This growing healthcare issue is costly in multiple aspects. Its financial burden may surpass that of managing the disease itself [14]. In Saudi Arabia, the medical health expenditures of people with diabetes are 10-fold higher than that of people without diabetes, which exerts an economic burden [15]. Furthermore, as suboptimal adherence may manifest as complications, it is the underlying cause of more than 30% of medicine-related hospital admissions [16]. Given the above, it is deleterious in terms of both health and economy [17].

Adherence and compliance are terms that are sometimes equated with one another and used interchangeably [18]. However, adherence is deemed superior to compliance, as it entails that the patient’s management plan was mutually tailored by the healthcare provider and the patient [19]. WHO defines adherence as “the extent to which a person’s behavior - taking medication, following a diet, and/or executing lifestyle changes - corresponds with agreed recommendations from a healthcare provider” [9]. Meanwhile, compliance is defined as “the extent to which the patient’s behavior matches the prescriber’s recommendations” [20]. Therefore, in this study, the term “adherence” will be mainly used.

Literature review shows a vast array of significant determinants of adherence [21]. These can be categorized into three major categories: the medical status [11], personal characteristics of the particular patient [22], and the agreed-upon therapeutic plan and medical encounter [9]. The personal characteristics contain the demographic data [22], forgetfulness [23], and personal beliefs [24]. The number of medications, complexity of the regimen, side effects, cost, and lack of trust in treatment efficacy are under the umbrella of the therapeutic plan and medical encounter [25].

The level of adherence among type 2 DM patients in Jeddah has been previously reported in a study [9]. However, there are some core differences between this study and the previous one: the earlier study was limited to the population of the national guard for health affairs and their three primary healthcare centers (PHCCs), whereas this study included PHCCs from all the clusters of the hospitals in Jeddah affiliated with the Ministry of Health, providing a better representative sample of the population of Jeddah. This is, to the best of the researchers’ knowledge, unprecedented. In addition, this study used a scale, rather than a yes/no question, to measure adherence [9]. This study also identified the determinants of suboptimal adherence, by correlating the variables with adherence using the multiple logistic regression model, ultimately placing them in a frame that allows their use as components of a prediction tool, for suboptimal adherence while interviewing patients.

## Materials and methods

Study area and setting

This study was conducted in Jeddah, which is the second-largest city in Saudi Arabia (population, ~3.4 million)[26]. The PHCCs in Jeddah conform to a geographical clustering system affiliating them to hospitals among five sectors: King Abdullah Medical Complex (11 centers), King Abdulaziz Hospital (six centers), East Jeddah Hospital (10 centers), King Fahad Hospital (13 centers), and Al-Thagher Hospital (six centers). Outpatient services pertaining to various medical needs across all ages and stages of life are provided at these centers. Services to patients with DM, hypertension, and asthma are provided by chronic disease clinics at these centers.

Study population

The study population included patients with type 2 DM who attended the Ministry of Health PHCCs in Jeddah.

Inclusion criteria

Eligible participants were consenting adults aged ≥18 years whose pharmacological management of type 2 DM was solely with OHAs and not with insulin. Participants who provided verbal consent were recruited for this study.

Exclusion criteria

Non-Arabic speakers were not recruited, as the questionnaire was in Arabic.

Sampling technique

Stratified random sampling was applied to the abovementioned sectors. The sample is proportional, and the selection process took place as follows: the centers in each sector were assigned numerical values as illustrated in Table 1. These values were entered in Random.org, and simple randomization was employed for each sector separately, resulting in a total of eight centers marked in Table 1.

**Table 1:** The Ministry of Health sectors and related primary healthcare centers in Jeddah * Due to the COVID-19 pandemic, some centers were assigned to serve only suspected coronavirus cases. Hence, they were not assigned a numerical value; they were eliminated from the randomization process. The Al Riyadh Center was also eliminated from randomization because it was closed. ** These are the eight primary healthcare centers selected by simple randomization.

Affiliated centers*			Hospital
Al Riyadh	2-Al Shaati	1- Thwal	King Abdullah Medical Complex
Obhur Al Shamalyiah	4- Al Wafa'	3- Dhahban**	
6- Alshiraa (505)	Al Rayyan	5- Al Hamdania	
	8- Specialized clinics	7- Khalid Al Namothaji**	
Ghulail	2-Al Qurayyat	1- Mada'in Al Fahad	King Abdulaziz Hospital
	4- Al Thaaliba	3-Al Balad	
	6- Al Qarinia	5- Al Mahjar**	
Al-Sulaimanyah	2-Al Jamieah	1-Briman	East Jeddah Hospital
5- Al Rabie and Al Tawfiq**	4- Al Rawabi	3- Raghamah	
7- Al Matar Al qadim	6- Al-Rehab	Guaizah	
8- Shraq Al Khati Al sarie**			
3- Al Azizia	2- Al Nahda	1- Al Ruwais	King Fahad Hospital
5- Al Salamah	4- Al Naeim	Al Hamraa	
8- Al-Safa (2)	7- Al Rabwa	6- Al Marwa	
11- Al-Bawadi	10- Al-Faisaliah**	9- Al-Safa (1)**	
12- Mushrifa			
3-Al Harazat	2- Kilo 14**	1-Prince Abdul-Majeed	Al-Thagher Hospital
Kilo 13	5- Om Al Silm	4- Al Muntazahat	

Data collection

**Figure 1:**
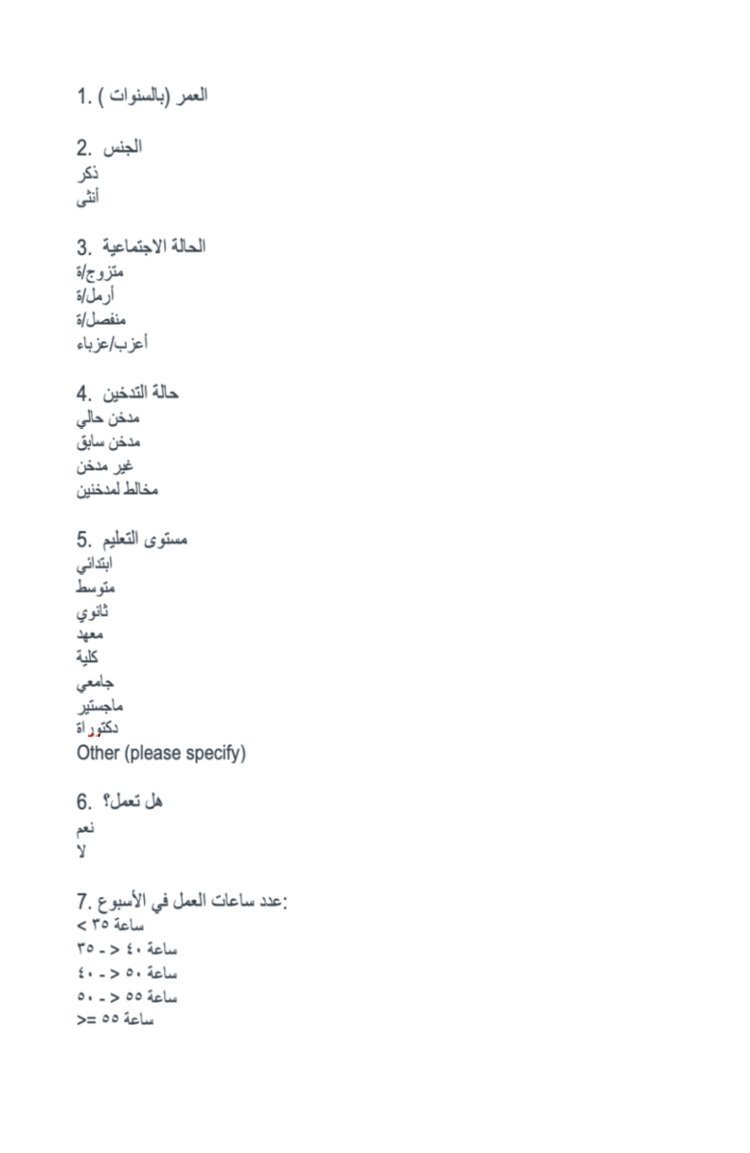
(A) The questionnaire form in Arabic

**Figure 2:**
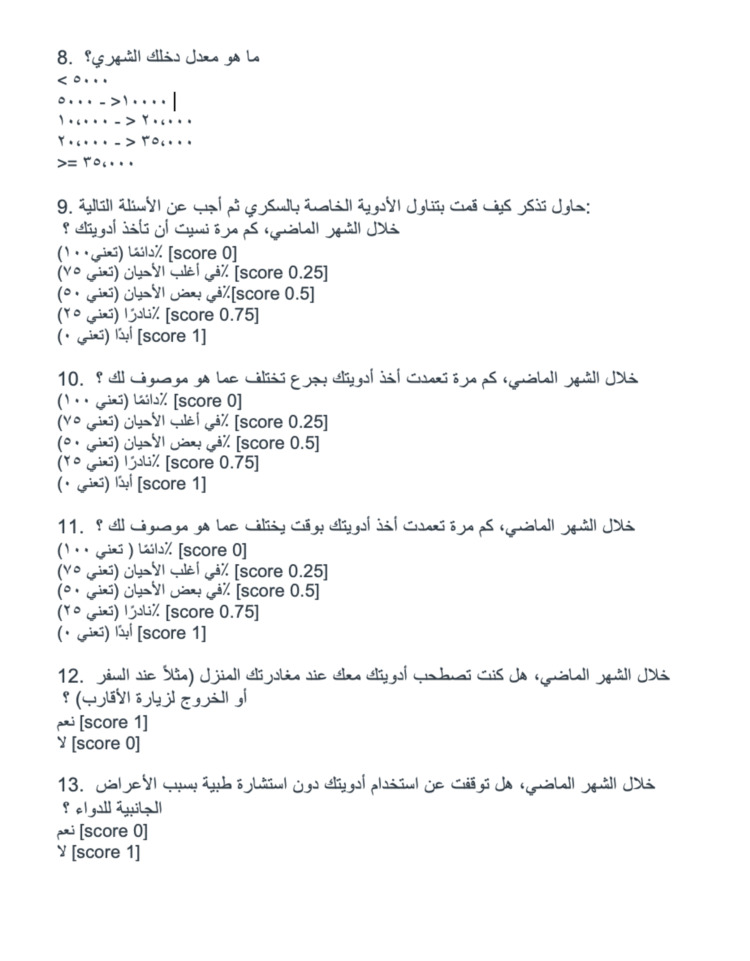
(B) The questionnaire form in Arabic

**Figure 3:**
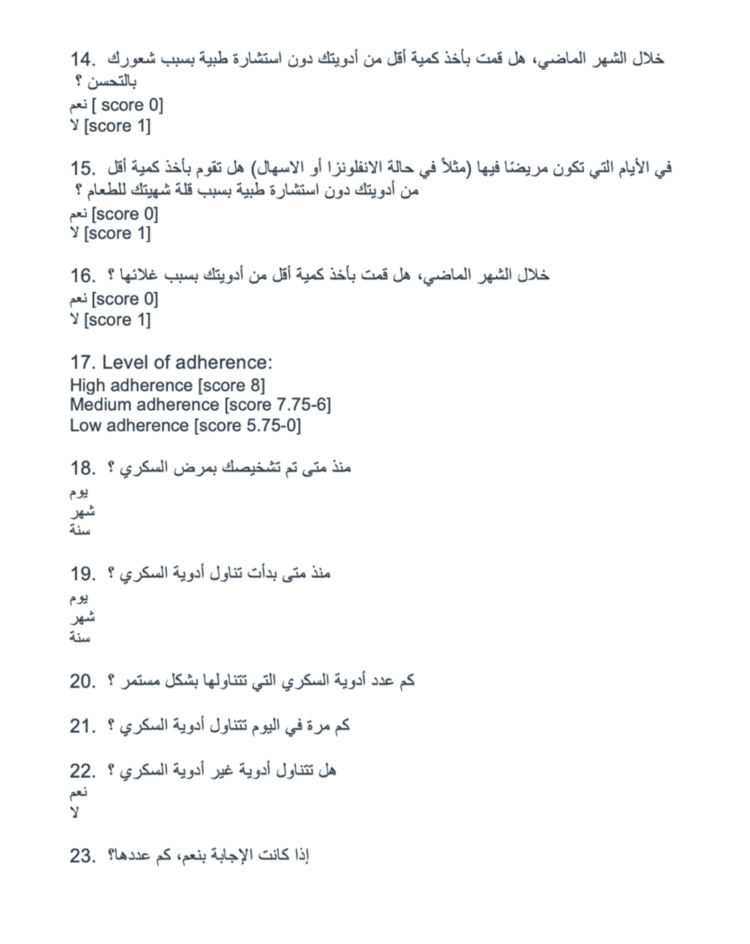
(C) The questionnaire form in Arabic

**Figure 4:**
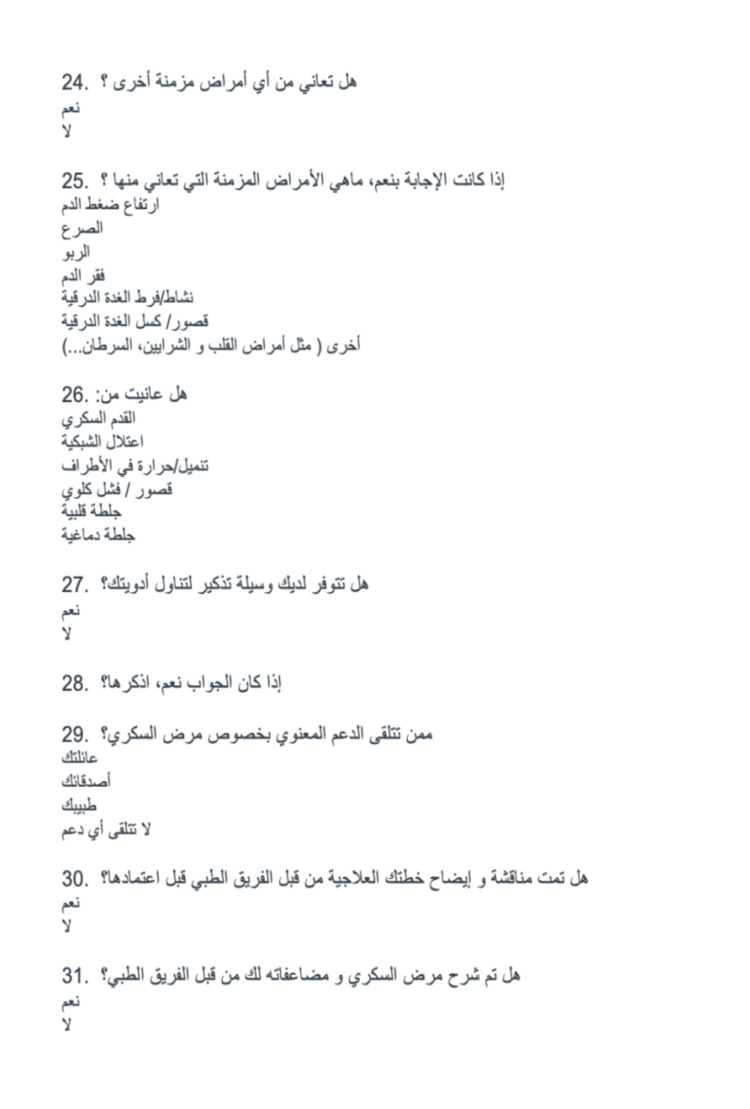
(D) The questionnaire form in Arabic

**Figure 5:**
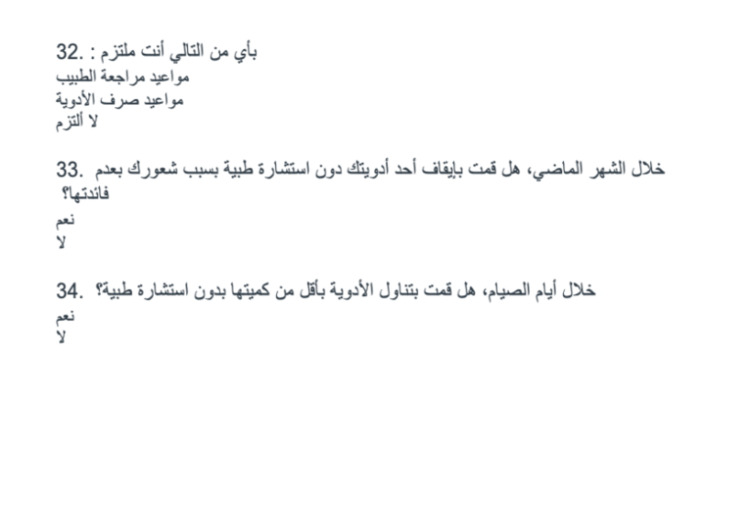
(E) The questionnaire form in Arabic

The instrument used was an electronic, self-constructed, validated questionnaire (Appendix 1: Figures 1-5). The questionnaire was in Arabic and contained three sections. The first section collected demographic data. The second section measured adherence using the Iraqi Antidiabetic Medication Adherence Scale (IADMAS) (Table 2). To the best of the researchers’ knowledge, there is no specific tool to measure the level of adherence to DM medication in the Arab population. However, a pilot study in Iraq formulated the IADMAS, which was validated and proven to be reliable, with a sensitivity of 100% and specificity of 33.9%. This scale has a statistically significant high correlation with the Medication Adherence Questionnaire of Morisky [27], which is the nearest to a gold standard for measuring medication adherence [28]. The last section of the questionnaire is a self-constructed section that assesses the determinants of adherence, which was developed based on a literature review.

**Table 2:** Determining adherence level by using the IADMAS* *A total score of 8 is classified as high adherence level, 7.75–6 is classified as medium adherence level, 5.75–0 is classified as low adherence level. **This item targets unintentional non-adherence. All other items target intentional non-adherence. IADMAS: Iraq Antidiabetic Medication Adherence Scale.

Item	Always	Often	Sometimes	Rarely	Never
1. During the last month, how many times did you forget to take your medication(s)?**	0	0.25	0.5	0.75	1
2. During the last month, how often did you take your medications deliberately in a dose different than what was prescribed for you?	0	0.25	0.5	0.75	1
3. During the last month, how often did you take your medications deliberately at a different time than was prescribed for you?	0	0.25	0.5	0.75	1
Item	Yes		No		
4. During the last month, did you take your medication(s) with you when leaving the house (as when visiting relatives or traveling)?	1		0		
5. During the last month, did you stop taking your medication(s) without seeking medical consultation because of side effects?	0		1		
6. During the last month, did you take lesser amounts of your medication(s) without seeking medical consultation because you felt better?	0		1		
7. During sick days (as in influenza or diarrhea), do you take lesser amounts of your medication(s) without seeking medical consultation because of decreased appetite?	0		1		
8. During the last month, did you take your medication(s) in lesser amounts because it was expensive?	0		1		

Data were collected between the end of November 2020 and the beginning of February 2021, throughout which the Corona pandemic occurred. With our targeted population being patients with type 2 DM, the interviews after the pilot study were resumed virtually through phone calls. Contact numbers were acquired from the chronic disease clinics, the daily visits records, and electronic databases, according to the infrastructure of the randomized center of interest.

Study design

This was an analytical, cross-sectional study.

Sample size planning

The sample size was calculated using the Raosoft website [29] with an accepted margin of error of 5%, estimated population size of 20,000, level of adherence of 64.3%, and confidence level of 95%. The calculated sample size was 347 patients. To compensate for defaulters and nonrespondents, 10% of the calculated sample size was added. Thus, the total sample size was 382 participants.

A pilot study using convenience sampling was conducted on 10% of the total sample, which is equivalent to 38 patients. These patients and centers were not included in the final sample.

Data entry

Data were entered through tablets, computers, and mobile devices into a SurveyMonkey database [30], from which it was exported in Excel format.

Data analysis

Responses containing missing data pertaining to the IADMAS or response errors were omitted. Hence, statistical analysis was carried out on 383 out of a total of 391 responses. The Statistical Package for the Social Sciences (SPSS®) version 20 (IBM Corp., Armonk, NY) was used for statistical analysis. Categorical variables were described by frequency and percentage, whereas continuous variables were described by mean ± SD. The total score of adherences was classified into two categories, optimal and suboptimal, according to the cutoff point. Chi-square test was employed to assess associations between adherence and categorical data. Multiple logistic regression analysis was employed to determine the predictors of adherence that were proved to be significant in the univariate analysis. The accepted level of significance was set below 0.05 (p<0.05).

Ethical consideration

Ethical approval for conducting this study was obtained from the research committee of the Joint Program of Family Medicine, in Jeddah, and the Ministry of Health’s Institutional Review Board (H-02-J-002), and the study was conducted in accordance with the principles of the Declaration of Helsinki. The confidentiality and anonymity of all participants were ensured. Verbal consent was obtained from each participant.

## Results

Sociodemographic and clinical characteristics of the participants

The mean age of the participants was 57.1 years (standard deviation, 10.6 years). Their ages ranged from 20 to 92 years. Most participants were males (53.3%), married (71.5%), and working (71.3%). About half (52.7%) had school-level education, whereas 35% had university-level education. The majority were prescribed two or less OHAs per day (67.6%). The frequency of medications was reported as twice per day by 35.3% and three times per day by 38.1% of participants. Having other chronic diseases was reported by 23.2% of the participants (Table 3). Regarding the total number of medications per day, 45.2% had more than five medications per day, and 38.6% had three to five medications per day.

**Table 3:** Sociodemographic and clinical characteristics of the participants CVA: Cerebrovascular accident

	n	%
Age (years)		
≤40	28	7.3
41–59	180	47.0
≥60	175	45.7
Gender		
Female	179	46.7
Male	204	53.3
Marital status		
Married	274	71.5
Not married	109	28.5
Working status		
Yes	273	71.3
No	110	28.7
Working hours per week (n = 110)		
≤40	68	61.8
>40	42	38.2
Level of education		
School	202	52.7
University	134	35.0
No	47	12.3
Smoking		
Yes	79	20.6
No	304	79.4
Duration since diagnosis with diabetes (years)		
≤5	173	45.2
6–16	158	41.3
>16	52	13.6
Other chronic diseases		
Yes	89	23.2
No	294	76.8
Type of chronic diseases		
Hypertension	172	44.9
Epilepsy	2	0.5
Asthma	23	6.0
Anemia	35	9.1
Hyperthyroidism	1	0.3
Hypothyroidism	35	9.1
Complications		
Retinopathy	65	17.0
Tingling extremities	167	43.6
Nephropathy	23	6.0
Myocardial infarction	17	4.4
CVA	21	5.5
Other medications		
No	64	16.7
Yes	319	83.3
Frequency of diabetes medications per day		
Once	51	13.3
Twice	136	35.5
Three times	146	38.1
Four times	50	13.1
Total number of medications/day		
<3	62	16.2
3–4	99	25.8
≥5	222	58.0

Level of adherence

High adherence was reported by 25.1% of the participants (n = 98), whereas medium and low adherence was reported by 56.1% (n = 219) and 17.8% (n = 68) of the participants, respectively. Suboptimal adherence was defined as medium or low adherence and was reported by 74.9% of the participants. A summary of the responses from which the level of adherence was determined is demonstrated in Table 4.

**Table 4:** Summary of responses to the direct adherence questions using the IADMAS IADMAS: Iraqi Antidiabetic Medication Adherence Scale

Item	Always (1)	Often (2)	Sometimes (3)	Rarely (4)	Never (5)
1. During the last month, how many times did you forget to take your medication(s)?	6 (1.6%)	16 (4.2%)	36 (9.4%)	101 (26.4%)	224 (58.5%)
2. During the last month, how often did you take your medications deliberately in a different dose than what was prescribed for you?	8 (2.1%)	10 (2.6%)	16 (4.2%)	28 (7.3%)	321 (83.8%)
3. During the last month, how often did you take your medications deliberately at a different time than was prescribed for you?	9 (2.3%)	21 (5.5%)	52 (13.6%)	77 (20.1%)	224 (58.5)
Item	Yes		No		
4. During the last month, did you take your medication(s) with you when leaving the house (as when visiting relatives or traveling)?	293 (76.5%)		90 (23.5%)		
5. During the last month, did you stop taking your medication(s) without seeking medical consultation because of side effects?	40 (10.4%)		343 (89.6%)		
6. During the last month, did you take lesser amount of your medication(s) without seeking medical consultation because you felt better?	72 (18.8%)		311 (81.2%)		
7. During sick days (as in influenza or diarrhea), did you take lesser amount of your medication(s) without seeking medical consultation because of decreased appetite?	69 (18%)		314 (82%)		
8. During the last month, did you take your medication(s) in lesser amounts because it was expensive?	22 (5.7%)		361 (94.3%)		

Factors associated in univariate analysis with suboptimal adherence

In univariate analysis, suboptimal adherence was significantly higher among those who were working, compared with those who were not [odds ratio (OR), 1.7; 95% confidence interval (CI), 1.1-3.0], those who did not have a reminder available (OR, 1.7; CI, 1.2-2.7), those who did not commit to refill of medications and/or follow-up appointments with a doctor (OR, 5.9; CI, 1.3-25.1), those who did not identify disbelief in the medication benefit as the reason for stopping their medications (OR, 2.1; CI, 1.1-4.1) (Table 5 and Table 6).

**Table 5:** Association between suboptimal adherence and sociodemographic and clinical characteristics of the participants OR: odds ratio; CI: confidence interval.

	Adherence		OR	95% CI	P-value
	Suboptimal n (%)	Optimal n (%)			
Age (years)					
≤40	24 (85.7)	4 (14.3)	2.5	0.8–7.7	0.099
41–59	140 (77.8)	40 (22.2)	1.4	0.9–2.4	0.108
≥60	123 (70.3)	52 (29.7)	Reference		
Gender					
Female	138 (77.1)	41 (22.9)	1.2	0.8–1.98	0.361
Male	149 (73.0)	55 (27.0)			
Marital status					
Married	209 (76.3)	65 (23.7)	1.3	0.8–2.10	0.336
Not married	78 (71.6)	31 (28.4)			
Working status					
Yes	90 (81.8)	20 (18.2)	1.7	1.1–3.0	0.045
No	197 (72.2)	76 (27.8)			
Working hours per week (n = 110)					
≤40	53 (77.9)	15 (22.1)	0.5	0.2–1.4	0.180
>40	37 (88.1)	5 (11.9)			
Level of education					
School	145 (71.8)	57 (28.2)	0.9	0.5–1.9	0.172
University	108 (80.6)	26 (19.4)	1.6	0.7–3.4	0.239
No	34 (72.3)	13 (27.7)	Reference		
Smoking					
Yes	61 (77.2)	18 (22.8)	1.7	0.7–2.1	0.600
No	226 (74.3)	78 (25.7)			
Duration since diagnosis with diabetes (years)					
≤5	130 (75.1)	43 (24.9)	1.0	0.5–2.1	0.983
6–16	118 (74.7)	40 (25.3)	0.9	0.5–2.0	0.964
>16	39 (75.0)	13 (25.0)	Reference		
Other chronic diseases					
Yes	65 (73.0)	24 (27.0)	0.9	0.5–1.5	0.643
No	222 (75.5)	72 (24.5)			
Other medications					
Yes	43 (67.2)	21 (32.8)	0.6	0.4–1.1	0.117
No	244 (76.5)	75 (23.5)			
Frequency of diabetes medications per day					
Once	42 (82.4)	9 (17.6)	1.8	0.7–4.7	0.218
Twice	100 (73.5)	36 (26.5)	1.1	0.5–2.2	0.835
Three times	109 (74.7)	37 (25.3)	1.1	0.6–2.3	0.712
Four times or more	36 (72.0)	14 (28.0)	Reference		
Total number of medications/day					
<3	49 (79.0)	13 (21.0)	1.2	0.7–2.6	0.709
3–4	74 (74.7)	25 (25.3)	1.1	0.6–1.8	0.871
≥5	164 (73.9)	58 (26.1)	reference		

**Table 6:** Association between suboptimal adherence and support among the participants OR: odds ratio; CI: confidence interval.

	Adherence		OR	95% CI	P-value
	Suboptimal n (%)	Optimal n (%)			
Available reminder					
No	211 (77.9)	60 (22.1)	1.7	1.2–2.7	0.040
Yes	76 (67.9)	36 (32.1)			
Commitment to refill appointments					
No	53 (77.9)	15 (22.1)	1.2	0.7–2.3	0.528
Yes	234 (74.3)	81 (25.7)			
Perceived commitment to medications refill and/or follow-up appointment					
No	32 (94.1)	2 (5.9)	5.9	1.3–25.1	0.007
Yes	255 (73.1)	94 (26.9)			
Family as a reported source of support					
No	150 (71.8)	59 (28.2)	0.7	0.4–1.1	0.117
Yes	137 (78.7)	37 (21.3)			
Friends as a reported source of support					
No	275 (74.5)	94 (25.5)	0.5	0.1–2.2	0.343
Yes	12 (85.7)	2 (14.3)			
Doctors as a reported source of support					
No	235 (73.4)	85 (26.6)	0.6	0.3–1.2	0.125
Yes	52 (82.5)	11 (17.5)			
Reported stopping because medications are not beneficial					
No	262 (76.6)	80 (23.4)	2.1	1.1–4.1	0.029
Yes	25 (61.0)	16 (39.0)			
Medical team explanation					
No	105 (75.0)	35 (25.0)	1.1	0.62–1.6	0.980
Yes	182 (74.9)	61 (25.1)			
Commitment to doctor appointment					
No	79 (79.8)	20 (20.2)	1.4	0.8–2.5	0.195
Yes	208 (73.2)	76 (26.8)			
Reported discussion of therapeutic plan with the medical team					
No	74 (71.8)	29 (28.2)	0.8	0.4–1.3	0.397
Yes	213 (76.1)	67 (23.9)			
Type of reminder					
Incorporating into daily routine	12 (57.1)	9 (42.9)	0.6	0.3–1.9	0.567
Family member/care provider/alarm	55 (67.1)	27 (32.9)	0.8	0.2–2.3	0.088
Organizers	8 (88.8)	1 (11.2)	Reference		

Factors associated in multivariate analysis with suboptimal adherence

In multivariate analysis, factors that predicted suboptimal adherence were as follows: no reminder available (OR, 1.9; CI, 1.1-3.1), no perceived commitment to medication refill and/or follow-up appointments with a doctor (OR, 6.1; CI, 1.1-3.1), and did not report stopping medications because medications were not beneficial (OR, 2.0; CI, 1.05-4.1). Age was inversely related to suboptimal adherence; suboptimal adherence decreased as age increased (p = 0.003). The total model was significant, and there was no multicollinearity. The model fitted the data (Hosmer test p-value, 0.567) (Table 7).

**Table 7:** Factors associated with suboptimal adherence in multiple logistic regression analysis OR: odds ratio; CI: confidence interval; B: regression coefficient.

	B	Adjusted OR (95% CI)	P-value
Age	-0.038	0.9 (0.93–0.98)	0.003
Available reminder			
No	0.625	1.9 (1.1–3.1)	0.017
Yes	Reference		
Perceived commitment to medications refill and/or follow-up appointment			
No	1.810	6.1 (1.4–26.5)	0.016
Yes	Reference		
Reported stopping because of disbelief in medication benefit			
No	0.702	2.0 (1.05-4.1)	0.038
Yes	Reference		

## Discussion

In its report on medication adherence, the WHO stated that “increasing the effectiveness of adherence interventions may have a far greater impact on the health of the population than any movement in specific medical treatment” [9]. Adherence is an ally of a better quality of life and overall health. Low adherence has been associated with inadequate glycemic control and increased rates of morbidity and mortality [9].

Studies from the eastern, central, and southern regions of Saudi Arabia have reported unanimous results of suboptimal adherence with the levels being 67.9%, 64.3%, and 89.3%, respectively [10-12]. In this study, the adherence level was no exception; the level of suboptimal adherence was 74.9%. These data offer a compelling invitation to pursue the roots of this issue and explore it further.

Previous studies showed contrasting results with regards to whether there was an association of adherence to age and gender. This study found that adherence is inversely associated with age, the younger the age, the higher the percentage of suboptimal adherence. This is in good agreement with the findings of a study done in Abha city, Saudi Arabia [22], and other studies done outside Saudi Arabia [5,31].

As for gender, studies performed in Gaza and Al Hasa showed a statistically significant association between female gender and higher levels of adherence [10,32]. In this study and in a study performed in the central region of Saudi Arabia, males reported higher levels of adherence, compared with females [17]. The p-value for gender reflects no statistical significance in this study. However, in the aforementioned study from the central region, there was statistical significance, which renders the statistical significance peculiarly varied [17].

The results substantiate previous findings in the literature that have identified the patient’s working status [25], beliefs about the consequences of diabetes medication [24], and having a reminder as being important determinants of adherence [33]. Most of the study participants relied on caregivers and alarms to remind them to take their medications.

As for the variable measuring the patient belief toward medications, in order to conform to the adherence definition, the question that was used to assess this variable further specified the behavior to be without the healthcare provider’s consult. However, belief in the beneficence of the medication intertwines with the trust in the healthcare provider. If the patients do not trust the healthcare providers, they will not consult them before stopping the medication that is not beneficial according to the patients’ belief, which leads to the therapeutic relationship being a potential confounder. Therefore, as accurate as this result might be for the specific question asked, it might not purely reflect the intended variable.

Forgetfulness [9], behavior-related factors (such as taking medications in different doses at different times), and side effects are well-documented influencers of adherence [34]. The study population reported that 41.5% forgot to take their medications at least once. Patients deliberately took their medications at different times and in different doses than was prescribed in 16.2% and 41.5% of cases, respectively.

The quality of the treatment relationship has been recognized as an important determinant of adherence [25]. An effective treatment relationship is characterized by an atmosphere in which alternative therapeutic means are explored, the regimen is negotiated, adherence is discussed, and follow-up is planned [9]. This is compatible with the results of the variable of general commitment to medications refill and/or follow-up appointments with doctors. The variable is significantly associated with a suboptimal level of adherence. In light of these results, it might be beneficial to introduce systems that alert clinicians to the irregularity or loss of follow-up, which keeps track of this indicator of potential suboptimal adherence. The results here delineated no statistically significant association between the discussion of the therapeutic plan and adherence, which could be related to cultural factors.

In this study, the effect of health literacy was measured in the form of explanation of type 2 DM and its complications by the medical team to the patients. Even though this result differs from that of some earlier studies [34], no significant association was noted between medical education and adherence to medication in this study. It is plausible that the level of health literacy, in regards to DM in Jeddah, is exceptionally high. A comparison of the studies measuring the level of knowledge about DM conducted in Jeddah [23] and adjacent areas, such as Al Riyadh [35], Makkah [36], Kuwait [37], and Al Ismailia in Egypt [38], showed that patients in Jeddah had more knowledge about DM, which lends support to this hypothesis.

In this study, the IADMAS was used to measure adherence. This is a subjective measurement, which could be perceived as a limitation in the sense of susceptibility to recall bias. However, the IADMAS limited the recalling interval to only one month, which is expected to minimize the risk of recall bias.

Adherence could be affected by the cost of medications, which was not included among the variables in this study. The Ministry of Health’s PHCCs provide free medications. Nevertheless, the cost is already very well documented as a determinant of adherence [39,40].

## Conclusions

The study found that the level of adherence is suboptimal among patients with type 2 DM in Jeddah. Suboptimal adherence to OHA results in an increase in mortality, morbidity, and financial burden. We recommend being extra vigilant when encountering an influencer or a predictor of suboptimal adherence while interviewing the patient. In addition, it might be prudent to utilize methods to further secure and augment adherence; thus, employing prevention to counteract possible suboptimal adherence.
